# Non-invasive vagus nerve stimulation: the future of inflammatory bowel disease treatment?

**DOI:** 10.1186/s42234-023-00129-y

**Published:** 2023-11-29

**Authors:** Bruno Bonaz

**Affiliations:** Service d’Hépato-Gastroentérologie, Grenoble Institut Neurosciences, Université Grenoble Alpes, Grenoble, France

**Keywords:** Vagus nerve, Vagus nerve stimulation, Non-invasive vagus nerve stimulation, Inflammatory bowel Diseases – pediatric

## Abstract

The vagus nerve regulates inflammation and cytokine release through the inflammatory reflex. Recent pilot clinical trials using implantable bioelectronic devices have demonstrated the efficacy of vagus nerve stimulation (VNS) in adult patients with inflammatory bowel diseases (IBD) as an alternative to drug treatments. However, the use of non-invasive VNS should be of interest in adults with IBD and even more in pediatric IBD. In this issue of Bioelectronic Medicine, Sahn et al. report that non-invasive transcutaneous auricular VNS attenuated signs and symptoms in a pediatric cohort with mild to moderate IBD thus opening new therapeutic avenues in the management of pediatric but also adult IBD patients.

Inflammatory bowel diseases (IBD) are chronic inflammatory disorders of the gastro-intestinal tract, represented by Crohn’s disease (CD) and ulcerative colitis (UC). Genetic susceptibility, environmental factors, and altered gut microbiota, leading to dysregulated innate and adaptive immune responses, are the core issue of IBD (Torres et al. 2017). Over 1 million residents in the USA and 2.5 million in Europe are estimated to have IBD, with substantial costs for health care. IBD can be diagnosed at any age but the majority of new diagnoses are made in adolescence and early adulthood (Loftus 2004). Since the middle of the twentieth century, the incidence of UC and CD has increased in the Western world (Kaplan 2015). There is no cure for IBD, medical treatment is only suspensive, and surgery is indicated in case of failure of medical treatment or complications but there is a recurrence of CD post-surgery. Anti-tumor necrosis factor (anti-TNF) therapy is the mainstay of IBD management, but requires indefinite continuation of treatment, and is associated with loss of response, and adverse events (Solitano et al. 2023). The expanding utilization of anti-TNF therapy combined with a continuous flow of newer biologics (anti-interleukins and anti-integrins) and small molecules (Janus kinase inhibitors, Sphingosine-1-phosphate receptor modulators) with high price contributes to the increase of medication costs (Burisch et al. 2023). In addition, 30-50% of patients are non-adherent to treatment (Chan et al. 2017).

Consequently, a non-drug therapy targeting anti-inflammatory pathways with no side-effects and a lower cost is of interest (Bonaz, 2021). One possibility is to use the anti-inflammatory properties of the vagus nerve (VN), the longest nerve of the organism, innervating all the digestive tract. Indeed, the VN inhibits the release of pro-inflammatory cytokines, such as TNF, through an interaction of acetylcholine on alpha7nicotinic receptors of splenic and gut macrophages, i.e. the inflammatory reflex involving vagal efferents (Pavlov et al. 2018). The VN has additional anti-inflammatory properties through vagal afferents by activating the hypothalamic pituitary adrenal axis. The VN also decreases intestinal permeability which is one of the mechanisms involved in the pathogeny of IBD (Bonaz 2022).

Bioelectronic medicine is based on neuromodulation of the nervous system restoring organ functions and health with less adverse effects than drugs (Olofsson and Tracey 2017). The VN may be stimulated either invasively (during a 1-h surgery) at the cervical level, with an electrode wrapped around the left VN, tunnelised under the skin and linked to a neurostimulator positioned under the left clavicle. Such an invasive VN stimulation (VNS), approved in the treatment of drug refractory epilepsy, has also been used with efficacy and no side effects for the treatment of adult CD in pilot studies (Bonaz et al. 2016; Sinniger et al. 2020, D’Haens et al. 2023). The three critical parameter settings of VNS are pulse width, frequency, and intensity. However, the optimal parameters of VNS to achieve efficacious inflammation-related symptomatic relief by recruiting the appropriate fibers within the VN are still unknown. Tsaava et al. (2020) reported in experimental conditions that specific combinations of pulse width, pulse amplitude, and frequency produced significant increases of the pro-inflammatory cytokine TNF, while other parameters selectively lowered serum TNF levels. In addition, the periodicity and duration of VNS is a matter of question between a continuous ON-OFF stimulation (Bonaz et al. 2016; Sinniger et al. 2020) and an electrical stimulation restricted to 1–4 times daily in sessions lasting 1–5 min (D’Haens et al. 2023), thus potentially limiting off-target effects such as hoarseness and discomfort but also saving battery power. The electrode used for invasive VNS does not stimulate all the VN and may stimulate non-appropriated VN fibers thus resulting in off-target effects. Indeed, in D’Haens study and ours, the VN was not completely encircled by the electrode and fibres not covered should require higher stimulation, whereas fibres located near the perineurium of a fascicle were exposed to a stronger electrical field (Helmers et al. 2012). Moreover, anatomical variations of the cervical VN can affect the responses of nerve fibres to electrical signals delivered through an electrode (Pelot et al. 2020). Selective VNS, such as fibre-selective or spatially-selective VNS, aims to mitigate this by targeting specific fibre types within the nerve to produce functionally specific effects (Fitchett et al. 2021). Finally, the cost of invasive VNS ranges from USD 30,000 to USD 50,000 (Badran et al. 2018a).

Thus, non-invasive VNS, not requiring surgical implantation of the device, is a safer and cheaper alternative. In particular, transcutaneous auricular VNS (taVNS) is of interest since the auricular branch of the VN innervates 100% of the concha of the auricle (Peuker and Filler 2002), and is afferent to the nucleus tractus solitarius, the entrance of the VN in the central nervous system. Thus, stimulating the concha is able to have anti-inflammatory properties by activating a vago-vagal reflex (Butt et al. 2020). Unlike implanted VNS, taVNS components are external. Electrodes are affixed to the ear at surface landmarks predetermined to target the underlying auricular branch of the VN. An external pulse generator delivers electrical stimulation to the adhesive or clipped ear electrodes, which can be portable, self-administered, and delivered at home (Badran et al. 2019). The only side effects seen are related to the administration of transcutaneous electrical current, which causes redness and skin irritation in some individuals at the site of stimulation (Redgrave et al. 2018). Non-invasive VNS stimulates the same brain loci than invasive VNS (Badran et al. 2018a). The locus coeruleus, the principal brain noradrenergic nucleus, directly connected to the nucleus tractus solitarius, is involved in the circuitry necessary for the anticonvulsant effects of VNS (Krahl et al. 1998). Wienke et al. (2023) very recently showed that taVNS systematically modulates behavioral, pupillary, and electrophysiological parameters of locus coeruleus-noradrenergic activity during cognitive processing and for the first time that the pupillary light reflex can be used as a simple and effective proxy of taVNS efficacy. These findings have important implications for clinical applications of taVNS. The VN projects to many brain regions involved in pain processing, which can be affected by VNS either invasive or non-invasive. In addition to neural regulation, the anti-inflammatory property of VNS may also contribute to its pain-inhibitory effects (Shao et al. 2023). Badran et al. (2018b) demonstrated that taVNS with higher pulse widths (250 µs and 500 µs), along with higher frequencies (10 and 25 Hz), have larger effects on activating the VN. Non-invasive VNS has shown anti-inflammatory effects both in experimental conditions (Go et al. 2022), as well as in healthy controls (Brock et al. 2017), and in clinical conditions (Wu et al. 2023; Drewes et al. 2021). As such, the use of non-invasive VNS should be of great interest in adults with IBD, while even more so in pediatric IBD where surgery is less feasible.

In this issue of Bioelectronic Medicine, Sahn et al. (2023) evaluated the efficacy and safety of ta-VNS in 22 pediatric patients with mild or moderate IBD with a fecal calprotectine (FC), a marker of intestinal inflammation, > 200 ug/g within 4 weeks of study entry. Subjects were randomized to receive either taVNS (with a pulse width of 300 µs and frequency of 20 Hz) or sham stimulation, 5 min once daily for 2 weeks, followed by a cross over to the alternative stimulation for 2 more weeks. At the end of week 4, all subjects received taVNS for 5 min twice daily until week 16. Primary study endpoints were clinical remission, and a ≥ 50% reduction in FC level from baseline to week 16. Heart rate variability recorded vagal tone and patient reported outcome questionnaires were completed during interval and week 16 assessments. At week 16, clinical remission was achieved in 50% of CD and 33% of UC patients. At week 16, 64.7% of those with a baseline FC ≥ 200 had a ≥ 50% reduction in FC. An 81% and a 51% median reduction in FC was observed in UC and CD subjects respectively. taVNS restored a normal vagal tone in patients with either a low or a high vagal tone on inclusion, as reported in our pilot studies (Bonaz et al. 2016; Sinniger et al. 2020). Thus, characterizing vagal tone before and after VNS, wether invasive or not, is of interest. TaVNS was safe, and no significant side effects were reported.

To the best of our knowledge, this is the first pilot study of non-invasive taVNS reported in pediatric IBD patients. This is a randomized study with active or sham taVNS, in contrast to the other studies performed invasively in adult IBD patients with no sham controls (Bonaz et al. 2016; Sinniger et al. 2020; D’Haens et al. 2023).

In conclusion, non-invasive VNS therapy, such as taVNS, opens new therapeutic avenues in the management of IBD, both in pediatric and also adult IBD patients. Of course, larger randomized double-blinded control study and, overall, a long-lasting follow-up of the patients are awaited with interest to confirm these promising results.

## Data Availability

Not applicable.

## List of abbreviations

CD: Crohn’s disease
FC: fecal calprotectin
IBD: inflammatory bowel diseases
taVNS: transcutaneous auricular vagus nerve stimulation
TNF: tumor necrosis factor
UC: ulcerative colitis
VN: vagus nerve
VNS: vagus nerve stimulation

## References

[CR18] Badran BW, Dowdle LT, Mithoefer OJ, LaBate NT, Coatsworth J, Brown JC, DeVries WH, Austelle CW, McTeague LM, George MS. Neurophysiologic Effects of Transcutaneous Auricular Vagus Nerve Stimulation (taVNS) via Electrical Stimulation of the Tragus: A Concurrent taVNS/fMRI Study and Review. Brain Stimul. 2018a May-Jun;11(3):492–500. 10.1016/j.brs.2017.12.009 PMC6487660 29361441

[CR26] Badran BW, Mithoefer OJ, Summer CE (2018). Short trains of transcutaneous auricular vagus nerve stimulation (taVNS) have parameter-specific effects on heart rate. Brain Stimul.

[CR21] Badran BW, Yu AB, Adair D, Mappin G, DeVries WH, Jenkins DD, George MS, Bikson M. Laboratory Administration of Transcutaneous Auricular Vagus nerve stimulation (taVNS): technique, targeting, and considerations. J Vis Exp. 2019;14310.3791/58984. 10.3791/58984 PMC6867597 30663712

[CR9] Bonaz B (2022). Anti-inflammatory effects of vagal nerve stimulation with a special attention to intestinal barrier dysfunction. Neurogastroenterol Motil.

[CR11] Bonaz B, Sinniger V, Hoffmann D, Clarençon D, Mathieu N, Dantzer C, Vercueil L, Picq C, Trocmé C, Faure P, Cracowski JL, Pellissier S (2016). Chronic vagus nerve stimulation in Crohn’s Disease: a 6-month follow-up pilot study. Neurogastroenterol Motil.

[CR7] Bonaz B, Sinniger V, Pellissier S (2021). Therapeutic potential of Vagus nerve stimulation for inflammatory Bowel Diseases. Front Neurosci.

[CR28] Brock C, Brock B, Aziz Q, Møller HJ, Pfeiffer Jensen M, Drewes AM, Farmer AD. Transcutaneous cervical vagal nerve stimulation modulates cardiac vagal tone and Tumor necrosis factor-alpha. Neurogastroenterol Motil. 2017;29(5). 10.1111/nmo.12999 27957782

[CR5] Burisch J, Zhao M, Odes S, De Cruz P, Vermeire S, Bernstein CN, Kaplan GG, Duricova D, Greenberg D, Melberg HO, Watanabe M, Ahn HS, Targownik L, Pittet VEH, Annese V, Park KT, Katsanos KH, Høivik ML, Krznaric Z, Chaparro M, Loftus EV, Lakatos PL, Gisbert JP, Bemelman W, Moum B, Gearry RB, Kappelman MD, Hart A, Pierik MJ, Andrews JM, Ng SC, D’Inca R, Munkholm P (2023). The cost of inflammatory bowel Disease in high-income settings: a Lancet Gastroenterology & Hepatology Commission. Lancet Gastroenterol Hepatol.

[CR20] Butt MF, Albusoda A, Farmer AD, Aziz Q (2020). The anatomical basis for transcutaneous auricular vagus nerve stimulation. J Anat.

[CR6] Chan W, Chen A, Tiao D, Selinger C, Leong R (2017). Medication adherence in inflammatory bowel Disease. Intest Res.

[CR13] D’Haens G, Eberhardson M, Cabrijan Z, Danese S, van den Berg R, Löwenberg M, Fiorino G, Schuurman PR, Lind G, Almqvist P, Olofsson PS, Tracey KJ, Hanauer SB, Zitnik R, Chernoff D, Levine YA. Neuroimmune modulation through vagus nerve stimulation reduces inflammatory activity in Crohn’s disease patients: a prospective open label study. J Crohns Colitis. 2023 Sep 21. 10.1093/ecco-jcc/jjad151 PMC10798868 37738465

[CR30] Drewes AM, Brock C, Rasmussen SE, Møller HJ, Brock B, Deleuran BW, Farmer AD, Pfeiffer-Jensen M (2021). Short-term transcutaneous non-invasive vagus nerve stimulation may reduce Disease activity and pro-inflammatory cytokines in rheumatoid arthritis: results of a pilot study. Scand J Rheumatol.

[CR17] Fitchett A, Mastitskaya S, Aristovich K. Selective neuromodulation of the Vagus nerve. Front Neurosci. 2021 May;24:15685872. 10.3389/fnins.2021.685872 PMC8180849 34108861

[CR27] Go YY, Ju WM, Lee CM, Chae SW, Song JJ (2022). Different Transcutaneous Auricular Vagus nerve stimulation parameters modulate the anti-inflammatory effects on Lipopolysaccharide-Induced Acute inflammation in mice. Biomedicines.

[CR15] Helmers SL, Begnaud J, Cowley A, Corwin HM, Edwards JC, Holder DL, Kostov H, Larsson PG, Levisohn PM, De Menezes MS, Stefan H, Labiner DM (2012). Application of a computational model of vagus nerve stimulation. Acta Neurol Scand.

[CR3] Kaplan GG (2015). The global burden of IBD: from 2015 to 2025. Nat Rev Gastroenterol Hepatol.

[CR23] Krahl SE, Clark KB, Smith DC, Browning RA (1998). Locus coeruleus lesions suppress the seizure-attenuating effects of vagus nerve stimulation. Epilepsia.

[CR2] Loftus EV (2004). Clinical epidemiology of inflammatory bowel Disease: incidence, prevalence, and environmental influences. Gastroenterology.

[CR10] Olofsson PS, Tracey KJ (2017). Bioelectronic medicine: technology targeting molecular mechanisms for therapy. J Intern Med.

[CR8] Pavlov VA, Chavan SS, Tracey KJ (2018). Molecular and functional neuroscience in immunity. Annu Rev Immunol.

[CR16] Pelot NA, Goldhagen GB, Cariello JE, Musselman ED, Clissold KA, Ezzell JA, Grill WM (2020). Quantified morphology of the cervical and subdiaphragmatic vagus nerves of Human, Pig, and rat. Front Neurosci.

[CR19] Peuker ET, Filler TJ (2002). The nerve supply of the human auricle. Clin Anat.

[CR22] Redgrave J, Day D, Leung H, Laud PJ, Ali A, Lindert R, Majid A. Safety and tolerability of Transcutaneous Vagus nerve stimulation in humans; a systematic review. Brain Stimul. 2018 Nov-Dec;11(6):1225–38. 10.1016/j.brs.2018.08.010 30217648

[CR31] Sahn B, Pascuma K, Kohn N, Tracey KJ, Markowitz JF. Transcutaneous Auricular Vagus nerve stimulation attenuates inflammatory bowel Disease in children: a proof-of-Concept Clinical Trial. Bioelectronic Medicine 2023 (in press). 10.1186/s42234-023-00124-3 PMC10583463 37849000

[CR25] Shao P, Li H, Jiang J, Guan Y, Chen X, Wang Y. Role of vagus nerve stimulation in the treatment of chronic pain. Neuroimmunomodulation. 2023 Jun 27. 10.1159/000531626 PMC10614462 37369181

[CR12] Sinniger V, Pellissier S, Fauvelle F, Trocmé C, Hoffmann D, Vercueil L, Cracowski JL, David O, Bonaz B (2020). A 12-month pilot study outcomes of vagus nerve stimulation in Crohn’s Disease. Neurogastroenterol Motil.

[CR4] Solitano V, Ma C, Hanžel J, Panaccione R, Feagan BG, Jairath V (2023). Advanced Combination Treatment with Biologic agents and Novel Small Molecule Drugs for Inflammatory Bowel Disease. Gastroenterol Hepatol (N Y).

[CR1] Torres J, Mehandru S, Colombel JF, Peyrin-Biroulet L (2017). Crohn’s Disease. Lancet.

[CR14] Tsaava T, Datta-Chaudhuri T, Addorisio ME, Masi EB, Silverman HA, Newman JE, Imperato GH, Bouton C, Tracey KJ, Chavan SS, Chang EH (2020). Specific vagus nerve stimulation parameters alter serum cytokine levels in the absence of inflammation. Bioelectron Med.

[CR24] Wienke C, Grueschow M, Haghikia A, Zaehle T, Phasic (2023). Event-related Transcutaneous Auricular Vagus nerve stimulation modifies behavioral, Pupillary, and low-frequency Oscillatory Power responses. J Neurosci.

[CR29] Wu Z, Zhang X, Cai T, Li Y, Guo X, Zhao X, Wu D, Li Z, Zhang L. Transcutaneous auricular vagus nerve stimulation reduces cytokine production in sepsis: An open double-blind, sham-controlled, pilot study. Brain Stimul. 2023 Mar-Apr;16(2):507–514. 10.1016/j.brs.2023.02.008 36801260

